# Using doughnut economics to structure whole-system thinking with multidisciplinary stakeholders – a soft systems approach

**DOI:** 10.1186/s42854-026-00093-1

**Published:** 2026-03-18

**Authors:** Annika Hjelmskog, Jaime Toney, Marian Scott, John Crawford, Cris Hasan, Petra Meier

**Affiliations:** 1 Social and Public Health Sciences Unit, School of Health and Wellbeing, University of Glasgow, Clarice Pears Building, 90 Byres Road, Glasgow, G12 8TB UK; 2 School of Geographical and Earth Sciences, University of Glasgow, Glasgow, G128QQ UK; 3 School of Mathematics and Statistics, University of Glasgow, Glasgow, G12 8QS UK; 4 Adam Smith Business School, University of Glasgow, Glasgow, G12 8QQ UK

**Keywords:** Climate change, Health, Wellbeing, Equity, Doughnut economics, Wellbeing economy, Soft systems, Sustainability

## Abstract

This study considers an alternative framework for sustainable and equitable human prosperity in the city of Glasgow. The city’s ambition to transform to a Green Wellbeing Economy is explored through cross-sector engagement with Doughnut Economics: a framework that considers social and ecological wellbeing simultaneously. Cities globally are home to an ever-increasing share of the world’s population, and disproportionate contributors to ecological degradation, making them crucial partners for progress towards this goal. Significant buy-in is therefore required from a diverse range of cross-sector and multidisciplinary stakeholders. We ask how conceptualising interconnections between people and planet is aided by the Doughnut framework, and whether it supports systems thinking for stakeholders working in policy and practice. This paper examines the potential of applying the Doughnut Economics framework in Glasgow, Scotland, to (1) enhance understanding of the socio-ecological systems we live in, and (2) guide action-oriented solutions with multidisciplinary stakeholders. We used soft systems approaches - a qualitative method that can help define complex problems and direct action - to navigate complexity, align plural perspectives, and identify enabling conditions for whole-system change. Drawing on five participatory workshops and desk-based analysis, we co-produced a City Portrait of Glasgow spanning 44 dimensions derived from the Doughnut framework. We find that the Doughnut framework encourages cross-sector collaboration, supports holistic systems thinking, and that the Portrait tools can identify policy synergies and trade-offs in relation to the Doughnut’s wellbeing goals. The framework therefore has the potential to promote policy coherence if these insights are subsequently translated and operationalised into meaningful city-wide action.

**Supplementary Information:**

The online version contains supplementary material available at 10.1186/s42854-026-00093-1.

## Significance

Practitioners and policymakers internationally are increasingly looking for and trialling approaches that go beyond ‘growth’ to bring social, ecological and economic value into decision-making. Doughnut Economics is being trialled in many cities to unite policies or service delivery, however, there have been very few scientific studies assessing how the framework can be fully operationalised. Previous studies have focussed mainly on its uses as a measurement tool and tracking social and ecological indicators simultaneously.

Societal adoption of Doughnut principles has the potential to represent a significant paradigm shift, going beyond intervening in metrics to transforming a mindset or worldview. To our knowledge, this is the first study to use a soft systems approach (in a real-world policy environment) to investigate the Doughnut’s potential to influence holistic understanding and therefore mindsets at the city scale.

**Supplementary Information:**

The online version contains supplementary material available at 10.1186/s42854-026-00093-1.

## Science highlights

The study provides a real-world application of systems thinking, with diverse stakeholder groups, to explore Doughnut Economics. It reveals complexity, synergies and trade-offs between multiple distinct but interconnected policy priorities, identifying possible co-benefits and unintended consequences from policies.

The Doughnut framework shows potential to both measure and guide city-based action towards sustainability. It also highlights where challenges may exist in implementation, e.g. misaligned spatial scales, and important policy levers held outside city governance frameworks. Stakeholders provided insights on the difficulty of judging a city’s performance against such a broad and often subjective set of wellbeing criteria.

Stakeholders from across the city system used the framework to incorporate and understand different perspectives. Our soft systems approach translated these perspectives into ‘conceptual models’ to inform future actions that may be more closely in line with Doughnut Economics principles.

This applied research explores how to lay the groundwork and create enabling conditions for whole-system transformation within the case study city. It has developed a collaborative and transdisciplinary foundation to support future applied research which will test how far these results can be turned into tangible, whole-system action.

## Policy and practice recommendations

• Secure support and buy-in for the Portrait’s vision from stakeholders across the system to adopt an ‘everybody, everywhere, all at once’ approach, uniting diverse, disparate actors around a shared, mutually-reinforcing goal.

• Maximise interdisciplinary and cross-sector expertise in policy and/or project teams to capture different views and encourage diverse insights. This may help create policies that are informed by richer understandings of the system they operate within.

• Utilise the four-lens framework to identify possible co-benefits and/or unintended consequences of city policies. Jointly apply sustainability and social justice principles to minimise negative trade-offs.

• Consider impacts of policies and decisions on the 44 dimensions (as derived from the Doughnut framework) as a whole, to guide holistic thinking and serve as a reminder for elements of social-ecological wellbeing that are most likely overlooked or not accounted for (such as the Global Social dimensions, i.e. Glasgow’s impact on the social wellbeing of citizens worldwide e.g. through supply chains).

• Design targets and indicators around the most influential leverage points, reflecting the complexity of the system they are designed to transform.

## Introduction and background

The Triple Planetary Crisis poses both direct and indirect threats to health, wellbeing, social and economic security (OECD 2025). Extreme weather events, unmanageable heat, widespread crop failure, infectious diseases, and forced migration will directly lead to adverse human outcomes (Rocque et al. 2021). Additionally, interventions to mitigate and adapt to these threats are likely to impact on wellbeing (Adger et al. 2022) in ways that, if poorly managed, could disproportionately exacerbate negative impacts for the most disadvantaged in society (Markkanen and Anger-Kraavi 2019). These indirect impacts may not always be immediate, but part of longer-term societal change, for instance, co-impacts related to economic growth of new sustainable industries or widespread redesign of low-carbon travel systems that do not consider affordability or accessibility. There is therefore an urgent need to balance ecological restoration with equitable societal transformation.

**Fig. 1:**
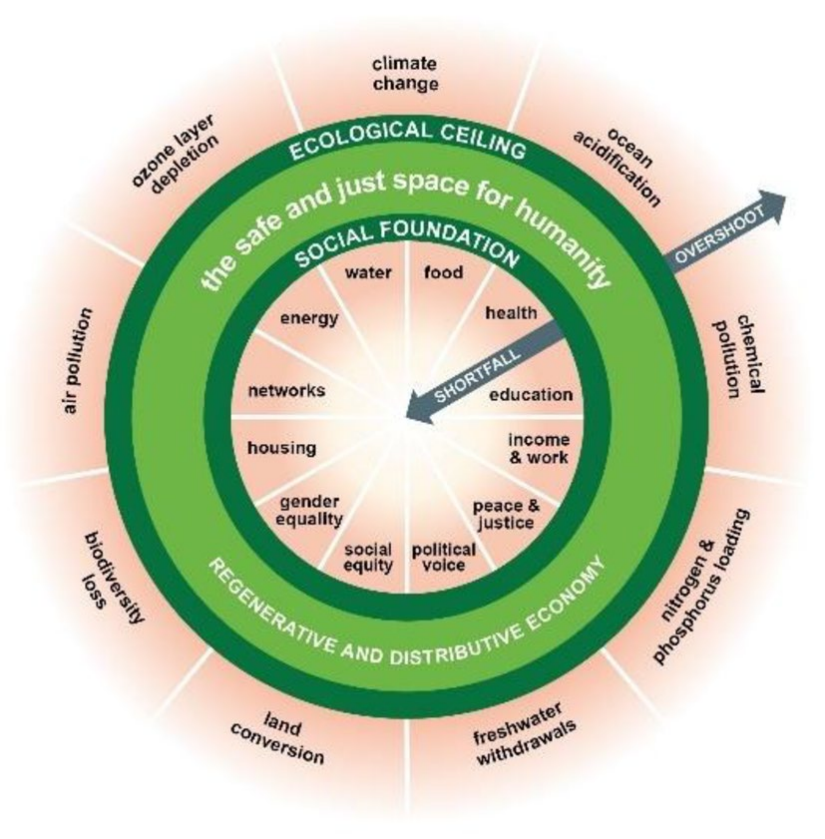
Title: the doughnut of social and planetary boundaries. credit: Kate Raworth and Christian Guthier. CC-BY-SA 4.0

One of the key challenges to achieving sustainable socio-ecological wellbeing is that prevailing mindsets, and accepted indicators of societal progress, tend to prioritise established measures of prosperity such as GDP (gross domestic product) (Costanza et al. 2009). Existing growth paradigms are recognised to have done little to achieve environmental sustainability or tackle social, health, or economic inequalities (Dixson-Declève et al. 2022). In response to this challenge, emerging alternative concepts include ‘planetary health equity’, which means ‘the environmentally sustainable and equitable enjoyment of good health’ (Friel 2022). Friel et al. (2022) suggest that systemic change is required to transform our current ‘consumptogenic’ system away from endless economic growth (with associated environmental degradation) and instead connect it with its corresponding resource extraction to achieve more holistic understandings of wellbeing that account for living within the planetary limits (Beddoe et al. 2009, Steinberger et al. 2020). Given this, a new paradigm that prioritises human and planetary wellbeing as its goal and measure of success is needed. The Doughnut Economics framework (Fig. 1) is presented here as an example of a possible future planetary health equity paradigm. It is one of several alternative approaches for urban development that take a more comprehensive and holistic approach to metrics of success, going ‘beyond GDP’ (Crisp et al. 2023, Khmara and Kronenberg 2023). Paradigms are ‘the sources of systems’ (Meadows and Sustainability 1999), and as Raworth (2018) argues in her presentation of Doughnut Economics:

‘*We have an economy that needs to grow*,* whether or not it makes us thrive. We need an economy that makes us thrive*,* whether or not it grows.*’ (Raworth 2018).

The Doughnut Economics framework (updated in 2025 (Raworth 2025)) is a conceptual model of human prosperity, based on two boundaries, one social and one ecological. It aims to balance a minimum social foundation (based on the Sustainable Development Goals) with a maximum pressure on the Earth’s natural resources (based on the Planetary Boundaries) (Rockström et al. 2009). The ‘safe space’ between the limits represents a future that meets the needs of all people within the means of the living planet. Since its initial publication in 2012, Doughnut Economics has inspired a growing body of research literature, with increasing publications engaging with its core concepts year on year (Lab and Academic articles 2020).

Given the size and growth of city populations, and the contribution of cities (particularly in affluent nations) to accelerating climate change (Negev et al. 2022, Hoornweg et al. 2020), radical change is required to transform cities sustainably (Moore et al. 2021). This change will require thoughtful design of climate responses to ensure that co-impacts are positive, and anticipate and prevent unintended negative consequences, including analysis of who benefits and who pays the costs (Oliveira et al. 2015, Sarkar and Webster 2017). Cities worldwide are experimenting with the Doughnut Economics framework (Raworth 2018) (Lab 2023) to make progress on the climate and wellbeing crises through action and innovation. The Doughnut framework contains an inherent demand for equity among global populations and more responsible global resource use, requiring a reduction in consumption practices that cause planetary harm by richer nations. As the relationship between inequality and climate change is shown to be two-way (Green and Healy 2022), achieving such a paradigm shift would represent the move from a vicious cycle of degradation and destitution to a regenerative cycle of socio-ecological wellbeing. The Doughnut framework is posited as suitable for embedding sustainable development in leadership, planning, and decision-making processes, due to its integration of ecological and social concerns (Turner and Wills 2022) and it may support policymakers to identify decisions that provide a greater propensity toward co-benefits with fewer trade-offs that could lead to improvements in urban governance, and more effective approaches to multi-dimensional wellbeing (Oliveira et al. 2015).

Delivering ‘win-win’ solutions to such complex, problematic challenges within a city will require a whole-systems approach, where parallel actions are taken in multiple places to affect the interdependent, emergent outcomes of social-ecological challenges (Gain et al. 2020). Such approaches recognise that complex, non-linear problems and challenges - in this case securing stable ecological conditions while supporting a thriving life for all people - require solutions that recognise the dynamic interdependencies inherent within the system (Reyers and Selig 2020). This asks policy- and decisionmakers to think differently, beyond the boundaries of their disciplines, expertise or direct areas of influence. There is an urgency to create this mindset shift alongside the urgency to decarbonise our global economies, where action is needed now. Winkelmann et al. (Winkelmann et al. 2022) argue that we need to reach beneficial ‘*social’* tipping points before we reach the negative ecological tipping points of the Planetary Boundaries (Wunderling et al. 2021), and that this necessitates generating the conditions for pushing policy regimes ‘into a qualitatively different state’ (Armstrong McKay et al. 2022) by triggering different ‘leverage points’ that may be at our disposal (Meadows and Sustainability 1999, Leventon et al. 2021). High on Meadows’s hierarchy of leverage points is the mindset, or paradigm, that the system is enabled by, superseded only by the ability to ‘transcend’ such paradigms to act and think in ways that are unbounded by them. Shifting mindsets, or worldviews, to a collective understanding that continued growth (for growth’s sake) is not conducive to improved wellbeing, could lead to a ‘regime shift’, transforming the way our institutions and technologies serve us (Beddoe et al. 2009, Leventon et al. 2021).

The goal of whole-system transformation would be for the component parts of the city system to be working in harmony, towards a state that is closer to the Doughnut’s ‘safe space’, rather than continued social shortfall and ecological overshoot (Fanning and Raworth 2025). Yet such an approach can be easier to support in theory than it is to deliver in practice (Morton et al. 2019), and a lack of progress can be attributed to ‘policy resistance’, which occurs when an inadequate understanding of a complex system results in missed opportunities and/or unintended consequences from policy interventions that may be well-intentioned but fail to consider their real-world systems context (Sterman 2001). Instead, we should aim for a state of ‘policy coherence’, which exists where policy interventions collectively integrate and manage system behaviours in a way that reinforces progress towards the overall goal for the system, so that ‘interventions trigger more policy synergies than conflicts’ (Castro 2022)(p. 414). A multi-sector effort may require transdisciplinary and transformative ways of working, to bring together those with responsibility for both social and ecological outcomes, with often diverse (or even contradictory) priorities, worldviews, and understandings of their place. This is in contrast to the siloed and linear approaches to policymaking that so often fail to account for interdependencies and complexities within a social-ecological system (such as a city) (Negev et al. 2022) (Oliveira et al. 2015, Lah 2025). To achieve this kind of coherence, scientific knowledge that is co-produced between practitioners and scientists and across multiple disciplines is required (Frantzeskaki et al. 2019, Lang et al. 2012). In this paper, we explore the potential of Doughnut Economics as a holistic approach to bring about synergistic co-benefits at the city-scale, enabling local people to discuss local and global framing of social and ecological challenges from a place-based lens and create agency for them to co-design alternative futures.

This study is part of a large, interdisciplinary research programme: ‘Glasgow as a Living Lab Accelerating Novel Transformation’ (GALLANT)(NERC), a City-University partnership to support Glasgow City’s ambitious plans to adopt Wellbeing Economy principles and achieve a just transition to NetZero by 2030 (Council). Prior to funding for GALLANT, Glasgow City Council and the University of Glasgow’s Centre for Sustainable Solutions co-developed a series of Green Recovery Dialogues, recognising the City’s recovery from Covid-19 as it headed toward hosting *COP26* as a key enabler for transformative change (Toney et al. 2025). These dialogues brought together researchers, practitioners and decisionmakers to reveal the complexity of Glasgow’s climate change challenges. The new City-University partnership was founded on a desire to adopt systems leadership, recognising the need to catalyse and empower collective action of others and strengthen coalition-building and collaborative leadership. Glasgow announced its intention to become a ‘Thriving City’ in partnership with C40 Cities during its hosting of *COP26* in 2021 (Council 2021).

### Research objective and contribution

The aim of this paper is to examine the potential of the Doughnut Economics framework in Glasgow, Scotland, as part of the city’s ambitions for whole-system transformation to achieve a state of ‘thriving’. The Doughnut framework may (1) increase complex understanding of the socio-ecological system we live in, and (2) guide or direct action-oriented solutions in a real-world setting, with large groups of multi-sector stakeholders, holding pluralistic views. We report results of downscaling Doughnut Economics to understand Glasgow as a socio-ecological city system, and discuss the potential of the Doughnut framework to drive and monitor whole-system transformation. The contribution of this study is threefold: empirical (the case study of Glasgow); methodological (incorporating the Doughnut into a Soft Systems Methodology); and conceptual (positioning the Doughnut as a systems thinking tool).

The paper is structured as follows: Introduction covers the background, the need for the study, and the research objective; Approach and Methods covers the City Portrait and Soft System Methodologies, and our desk-based and participatory processes; the Results section shares our experiences of the workshops, gives examples of policy-based exercises, and includes the full set of 44 co-created Thriving Definitions; and our Discussion and Conclusion interprets these findings in the context of whole-system thinking, and the limitations/future directions of the research.

## Approach and methods

### Thriving city portrait - applied soft systems methodology

**Table:** 

**Fig. 2:**
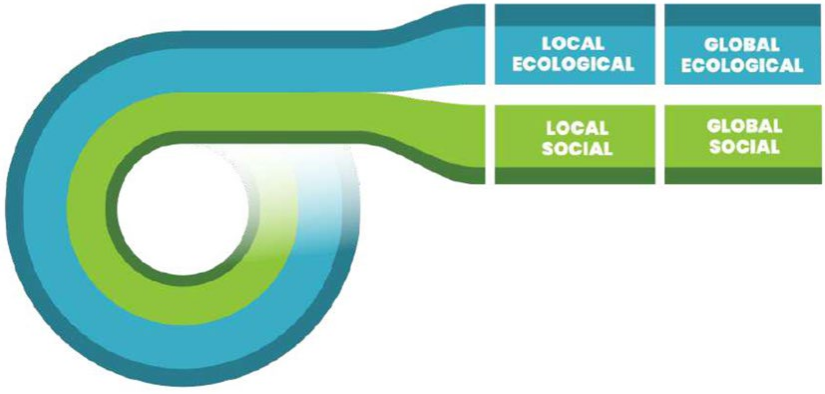
The ‘unrolled’ doughnut derived from DEAL - doughnuteconomics.org

**What is a City Portrait?** ^1^
A downscaled interpretation of the Doughnut Economics framework at the level of the city. A City Portrait provides a holistic snapshot of the city, asking what it would mean for a place to exist within the Doughnut’s boundaries (and how it could get there). The portrait is intended to inspire whole-system thinking and long-term transformation in a unique and locally relevant way.
Glasgow’s Thriving Portrait is therefore designed to answer the question:
*‘How can Glasgow become a home to thriving people in a thriving place, while respecting the wellbeing of all people and the health of the whole planet?*
The answer to this question is shaped by ‘Unrolling the Doughnut’ (Fig. 2) and breaking it down into four distinct but interconnected ‘lenses’ (see also Fig. 4). A Thriving City is therefore one that exists in the Doughnut’s ‘safe space’ for all four lenses.

^1^Doughnut Economics Action Lab (DEAL) have since substantially modified their methodological guide to provide a set of ‘Doughnut Unrolled’ tools aimed to support places engaging with the Doughnut framework at a downscaled level: ‘Doughnut Unrolled’ - Now in Five Languages | DEAL.

This research responds to a specific challenge in Glasgow: the Council’s intention to reorient the city system towards ‘wellbeing’ (social *and* ecological) in a way that brings it closer to the ‘safe space’ of the Doughnut. This will require holistic action, corralling diverse stakeholders from multiple sectors and departments around a shared, mutually beneficial/reinforcing goal. For social practitioners to think in ecological terms, and vice-versa, represents a departure from the traditionally siloed or departmentalised ways of working in a city administration. The goal is to design and enact actions and policies that will improve on the status quo across the entire system. Therefore a soft systems approach (Armstrong 2019, Checkland et al. 2020) was selected as it is well-suited for problematic ‘real world’ situations (such as this challenge of improving socio-ecological wellbeing in a city).

In Soft Systems Methodology (SSM), a rich, qualitative approach to defining the problem allows for understanding of the inherent complexity and dynamism of human systems. Soft systems approaches are designed to build systematic understanding of complex, non-linear, and ill-defined situations as part of a research process that will ultimately drive *action* (Höhn et al. 2023). For this reason, we selected SSM for the Portrait rather than, say, a Participatory Action Research (PAR) approach (which has the benefit of engaging and empowering community members to drive social change, but does not have the same focus on finding common ground for improvement (Cornish et al. 2023). The iterative process of building collaborative solutions in SSM can accommodate the pluralistic understandings and preferences that are held by diverse stakeholders, and securing broad stakeholder buy-in to the goals of the framework is a key ambition of this work (Höhn et al. 2023, Checkland 2000). The pressing need to develop actionable insights made SSM more appropriate for this work than other methods of complex system mapping, or modelling, which may have offered more advanced ways to understand the system, but have less focus on bringing the necessary stakeholders on board throughout the process. SSM is designed to ensure that human concerns and nuanced, valuable understandings and perspectives about the system are incorporated early in the process (Kirk 1995). Additionally, its cyclical nature allows for different speeds and degrees of transformation, foreseeing that only incremental shifts, or action from ‘early adopters’, may be more likely during the initial stages of engagement with the framework.

**Fig. 3:**
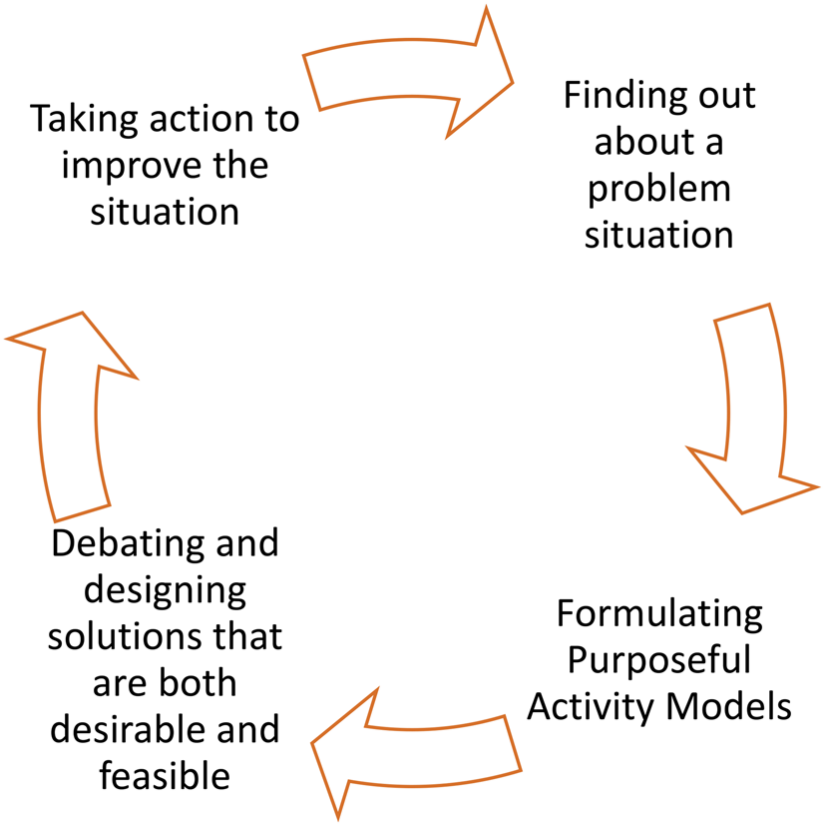
Checkland’s soft systems methodology and the city portrait

The stages of SSM are shown in Fig. 3. In this study, we address the first two stages in detail, laying the groundwork for the future stages of this applied research. We combine the principles of SSM with the pilot methodological approach of our collaborators at C40 Cities^2^ and Doughnut Economics Action Lab (DEAL) for creating ‘City Portraits’ (Initiative and CREATING CITY PORTRAITS - 2020). This research therefore represents an experimental approach, co-produced in stages with transdisciplinary partners. The principles of SSM provide theoretical grounding and structure to the practical, iterative process of designing a ‘thriving’ future for Glasgow.

We drew on our partners’ (C40 Cities and DEAL) guidance for ‘Unrolling the Doughnut’ to downscale the global principles of the Doughnut to a local scale^3^. This loose methodology requires the resource and work for this downscaling to be led and carried out locally, which in Glasgow was made possible through the City and the University agreeing to co-lead and share the resources needed to create the Portrait (this is likely to be a stumbling block early on for many places). The approach incorporates several principles of Soft Systems Methodology to support implementing the change process, including the early involvement of stakeholders through co-production, acknowledgement of complexity and interconnectedness, and a responsive awareness of local context (Augustsson et al. 2019). We have therefore applied SSM here to bring rigour and structure to an otherwise relatively open and heuristic process. The suggested methodology incorporates a combination of desk-based research, stakeholder engagement and participatory workshops and supports cities to take a holistic view and create new perspectives on what it means for that place to thrive. The activities address questions on the role and potential of Glasgow to thrive across the wider social-ecological system, both for local benefit and worldwide (Fig. 3):

**Fig. 4:**
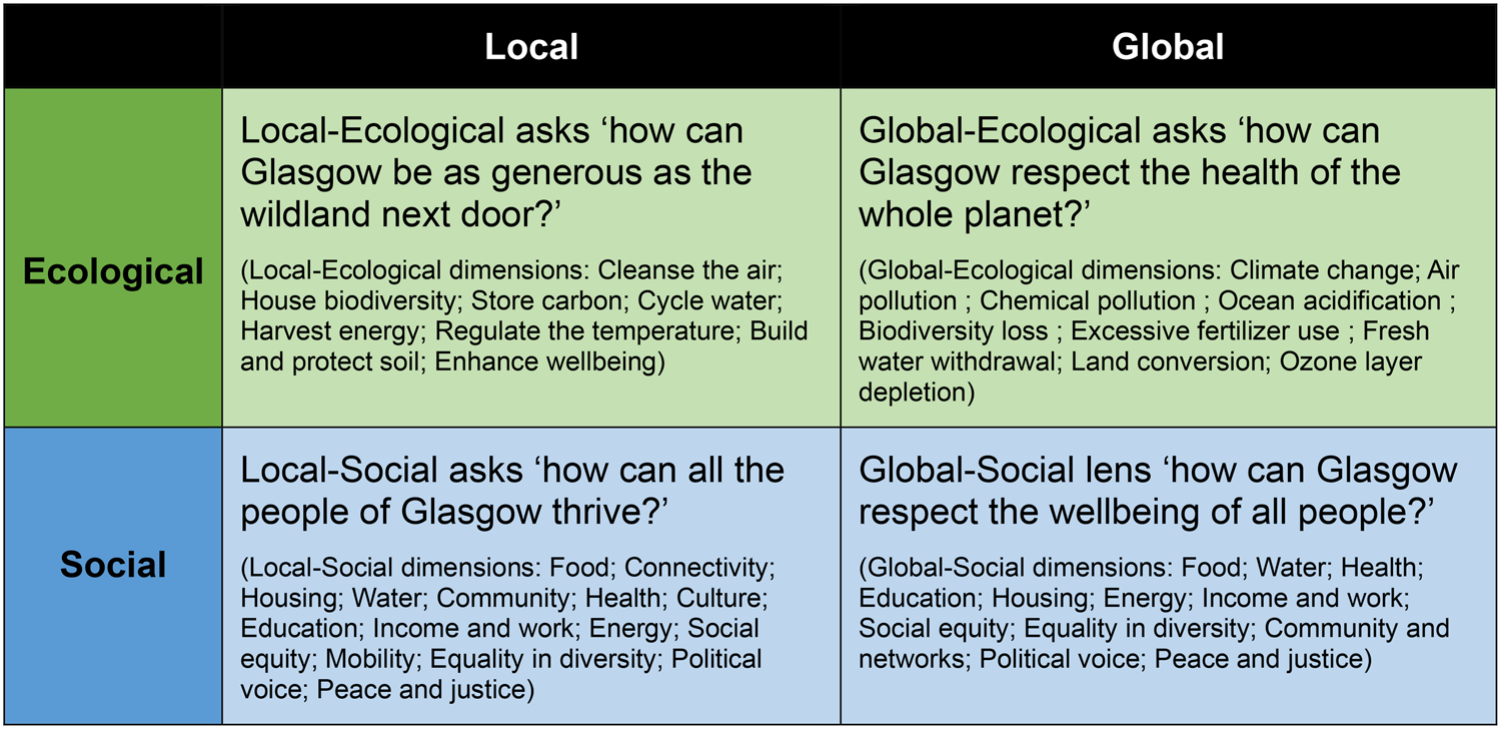
The four lenses of the ‘unrolled’ doughnut

The full Portrait comprises 44 dimensions across the four lenses (see Fig. 4). The dimensions in each lens are broadly the same as the inner and outer dimensions of the original Doughnut framework, but with some adjustments made for local dimensions (such as the addition of ‘Culture’ and ‘Mobility’ in the Local-Social lens, and the adaptation of Local-Ecological dimensions to reflect the question of mirroring nature’s generosity e.g. ‘Cleanse the Air’, ‘Harvest Energy’ etc.) We interrogated these dimensions through our combination of desk-based research and stakeholder workshops, through a process of activities designed to contribute to the cycle of applied learning that can drive real world action (see Fig. 3; Table 1) (Checkland 2000, Checkland and Scholes 1999).

As described in Table 1, the first step concerns defining an ambitious goal or challenge, in consideration of a problem that needs solving (Checkland and Scholes 1999). In this case, the initial task was to improve our understanding of Glasgow as a city system in relation to the Doughnut framework, and use this understanding to collaboratively define a shared vision of what ‘Thriving’ would look like in the Glasgow context.

**Table 1:** Applying SSM to the Glasgow case study

Activity	Application in the Glasgow case study
Finding out about a problem situation	• **Reviewing the context**. Glasgow City leaders have set 2030 as the target date for achieving NetZero and committed to becoming a Green Wellbeing Economy [51] that prioritises social and ecological wellbeing and tackles entrenched inequalities. These commitments require the support of a large, diverse group of stakeholders with different worldviews, and complex solution spaces will require careful negotiations between priorities, trade-offs, unintended consequences, and uncertainty about the future. • **The problem situation is expressed**. The desk-based (preliminary) portrait contributed to understanding the current state of the system, from a systems perspective looking for alignments between existing strategies and targets. The process created a ‘picture’ of Glasgow by outlining the current breadth and focus of city policy, while highlighting some of the ways current efforts are separated by function or department, and identifying some existing indicators.
Formulating purposeful activity models	• Through the workshops and engagement activities, ‘Root Definition(s)’ of relevant systems were created, leading to: • ‘Conceptual models’ which define what a thriving Glasgow *could* look like. These are Glasgow’s ‘Thriving Definitions’ (see *Results*). These were agreed upon while simultaneously discussing the state of current relevant systems and what might therefore need to change.
Debating and designing solutions that are both desirable and feasible	• Iterative, solutions-focussed action pathway workshops, resulting in proposed solutions (collaborative work between C40 Cities, Glasgow City Council, and the individual GALLANT work packages, alongside supporting GALLANT research focussed on developing indicators, system mapping, policy mapping and quantitative modelling) *This is the scope of future work.*
Taking action to improve the situation	• *This will form part of the subsequent stages of work*,* and will inform iterative*,* ongoing evaluation that will be the subject of future publications.*

### Desk-based portrait (finding out about the problem situation)

Desk-based research to produce what the Doughnut Economics Action Lab call a ‘Data Portrait of Place’ (Lab and Doughnut Unrolled 2022) consisted of reviewing more than twenty published city policies and strategies to identify existing targets or ambitions that relate to different dimensions of the Doughnut framework e.g. (Council 2022, Council 2018, Council 2022, Council 2020, Partnership 2021, Region 2019). This provided an initial understanding of what ‘thriving’ might mean for the city on existing terms. To make an assessment about how near or far the city currently is from its ambitions, suitable indicators are then selected from publicly available data and statistics, and a comparative assessment is made between the ambitions, and the current state of performance. The most appropriate targets and indicators are then assigned to each of the Portrait dimensions, to provide an overview (see Supporting Information). The desk-based Portrait is based on targets and indicators of ‘best-fit’, often only offering narrow interpretations of the dimensions which are more complex in reality, and interconnected. The workshop process is designed to enrich this holistic ‘snapshot’ and draw focus to the system dynamics which may be highlighted by considering the 44 dimensions in their whole-system context.

### Engagement and workshop activities (formulating purposeful activity models)

The second stage of research formed the majority of our activity and explored the four-lens framework with expert stakeholders through in-person engagement and workshop activities. The purpose of this was to embed the Doughnut framework with a broad cross-section of city stakeholders, and build a richer, more complex vision of a ‘Thriving’ Glasgow that uses the holistic local-global understanding of the Doughnut to collaboratively assess the city system. We undertook a series of five workshops, each with different stakeholder groups, totalling approximately 130 participants. Participants were invited following a process of collaborative, iterative identification between the researchers and City Council partners (based in Glasgow City Council’s Sustainability Team), who have detailed, grounded knowledge of the relevant stakeholder groups and active organisations across Glasgow. Participants were selected based on their experience or expertise in one or more of the Portrait lenses, and where possible, each workshop’s list of invitees was designed to collectively cover social, ecological, local and global perspectives.

Workshops consisted of participatory discussions around the state of the current system, and what a thriving city could look like, with separate discussion groups initially considering the domains of one of the four lenses. Participants were then asked to identify relationships and to map connections between the different dimensions across the four lenses. The workshops were designed through collaborative discussions with the interdisciplinary research team and co-facilitators from Glasgow City Council. Depending on the audience, we consulted relevant staff (e.g. Cities and Regions Lead, Communities and Education Lead) at DEAL to cross-check workshop design with their principles and experience of translating the framework into practice.

Participants were asked throughout the workshops to increasingly engage with more of the unrolled doughnut framework, starting from a singular perspective (such as local-ecological, or global-social) and responding to the question of what ‘thriving’ would look like in that lens. Next, they were asked to consider how the dimensions of their starting point lens were connected to dimensions of the other three, and to identify and describe some of the key interactions. Finally, participants used the four-lens framework to consider Glasgow-relevant policy priorities from the four interconnected perspectives, to encourage whole-system, holistic understanding of the issues.

Following the workshops, the principal researcher digitally transcribed the content of the worksheets and categorised the suggestions for ‘thriving’ by both lens and individual dimension. Analysis was undertaken collaboratively and iteratively by the wider research team, and suggestions from the participants were condensed, amalgamated, and cross-referenced to generate single, coherent definitions for each dimension. Further details and suggestions that were also present in the data were incorporated into a more detailed description of ‘what this could look like’ to highlight enabling factors and conditions that participants identified as important for making progress. These definitions and enabling factors were then sense-checked with the Council partners in two follow-up consultations, and amendments were made until all partners were confident that the content reflected the discussions they had facilitated during the workshops.

The series of structured questions posed about the problem situation were designed to lead to ‘the emergence of a structured and coherent debate about intended change’ (Checkland and Tsouvalis 1997)(p.153) and therefore the accommodation of multiple perspectives around a unifying end goal. The conclusions of the discussions are therefore intended to inform a series of subsequent action pathways that will contribute to iterative cycles of improvement. In this study, the methods are intended to support co-production of research with the City Council, and to maintain the important emphasis on purposeful activity, implementation, and actionable insights, so that the research does not take place in an academic vacuum but as part of the city’s real-world policy context.

## Results

The aim of the Portrait process was to encourage stakeholders to think in more holistic terms about their spheres of influence, in terms of both the current system, and future action. We also assessed whether and how the Doughnut framework added particular value to this exercise in systems thinking. The output is the ‘Thriving Glasgow Portrait’: a multi-dimensional answer to the question of what it would mean for Glasgow to thrive, representing voices from across the city system. This whole-system definition of ‘thriving’ takes the form of forty-four co-produced definitions, one for each of the dimensions included in the four lenses (see Table 2). Through the contributions of stakeholders with diverse areas of knowledge and expertise, the full range of both social and ecological factors were discussed in the various group settings. Our results are presented in both the co-created definitions themselves, as well as in the insights from the collaborative, cross-system process that included multi-sector workshops.

### Desk portrait of city strategies

The initial desk-based research (see Supporting Information) highlighted that many of the city’s existing policies and strategies were concerned with one or more of the Doughnut’s dimensions, but these dimensions were frequently considered separately, rather than as interconnected parts of an overarching wellbeing ambition. The evident siloed nature of policymaking highlighted the current lack of a coherent, cross-system framework to guide city action. Additionally, some strategies contained either gaps or contradictions in terms of the goals of the Doughnut, and in some examples the city’s social and ecological ambitions could be viewed as in conflict. Using the Doughnut to assess these dimensions simultaneously highlighted strategy areas that are therefore likely to lead to policy resistance. For example, the 2022 Glasgow Transport Strategy (Council 2022) aims to prioritise decarbonisation, but refers to Glasgow Airport only in terms of increasing its (sustainable) local transport links, thereby making aviation easier and boosting tourism. This is despite also referencing a national ambition to ‘decarbonise scheduled flights within Scotland by 2040’ (p.18). Flights within Scotland account for only a small proportion of the CO_2_ emissions associated with Glasgow Airport, however, and the date of 2040 is a full decade later than Glasgow’s ambition to become net zero by 2030, indicating a lack of both spatial and temporal alignment between the scale of the target and the systemic context of the issue.

The Doughnut framework was useful for structuring the existing assessment of ‘Thriving’ in the city (which exists in a dispersed collection of policy and strategy documents) around a coherent framework. The four-lens framework also places equal value on each of the forty-four dimensions, which helped to highlight how/where most political energy is currently placed, or where current knowledge or data collection is available to support progress towards certain goals – as well as shining a light on parts of the framework that appear to be either overlooked, or more challenging to progress. For example, the desk-based review highlighted that in the Local-Social lens, no suitable targets could be identified for the ‘Peace and Justice’ dimension. The greatest number of gaps emerged in the Global-Social lens, where several domains had no obvious targets against them (for example, Housing, Health, Political Voice). The latter illuminated how little the issue of a city’s influence on global wellbeing (through supply chains, material extraction etc.) is understood, considered and given prominence in local decision-making. The presence of all forty-four dimensions in the framework at the same time, or on the same diagram, was both a strength and a challenge. It covers such a large range of policy areas that there is a risk of overwhelming those engaging with the framework when ‘everything matters’, but it also drew consistent attention to gaps in our knowledge that might otherwise have been easy to minimise or disregard. The desk-based Doughnut therefore helped to identify some of these potential policy synergies, conflicts, and gaps in preparation for more detailed discussion in the workshops.

### Stakeholders’ perspectives on thriving

Two key observations are that firstly, irrespective of stakeholder starting point (in terms of background, or discipline), workshop participants were able to use the Doughnut Economics four-lenses framework to engage with and contribute insights into parts of the system that would have been less or un-familiar to them beforehand. As an intuitive conceptual tool that enables systems thinking for planetary health, the Doughnut appears to have significant potential. Secondly, many of the suggestions for what a ‘thriving’ Glasgow could look like were shared, repeated and reinforced across different workshops, suggesting that there is significant potential for a shared vision, and alignment across goals and priorities that accommodate perspectives from a wide range of stakeholders, thus laying the groundwork for whole-system policy action that is both ‘acceptable’ and ‘feasible’. Despite the diverse backgrounds of workshop participants, there were fewer incidences of contentious or conflicting views causing tension during this process than anticipated. Observations from facilitators suggest that this may have been due to the future orientation of the questions that we asked people to consider, creating a safe space for ideation, and respectfully having a “parking lot” for discussions that took us away from the main aims of the day, as being key factors that limited conflict.

The Doughnut was effective for retaining the holistic systems overview that connects the different, diverse stakeholders who might not usually see how their areas of work and expertise align or intersect (for example, representatives from the city’s Chamber of Commerce in discussion with experts on Soil Health). At the start of the workshops, it had been made clear that given the scope of the Doughnut Economics framework, no individual was expected to have insights across all four lenses, or for the majority of dimensions. Instead, participants were seated in groups to reflect the spread of attendees’ backgrounds, and facilitators encouraged participants to share their knowledge during group discussion, allowing for whole-system insights and areas of connection to emerge. The systematic approach to breaking down the Doughnut framework gave structure to each lens and served as a visual reminder of the social or ecological elements that are deemed to be important for thriving. It was still a challenge to cover the full spread of dimensions within a single lens during the discussions, however, and facilitators used a ‘Park It’ worksheet to record issues that participants found difficult to reach consensus on, or which took the conversation away from the main objectives of the session (for example, participants demonstrated complex views debating ‘green growth’ vs. ‘degrowth’). This both steered and shaped discussion, as well as creating space to highlight areas that received little or no attention (for example, very few suggestions were made relating to ‘Connectivity’ in the Local-Social lens).

The variation in the participants’ backgrounds, as well as the huge ambition of the Doughnut framework, did present some challenges for workshop design and facilitation. In order to be equitable and democratic, the Doughnut invites diverse responses, and our intention was to represent a very broad cross-section of Glasgow in the process and finalised Thriving Definitions. To counter the possibility of overwhelm or confusion that might occur when faced with forty-four dimensions to consider, we gave individual groups only one lens to discuss in detail. The groups were decided in advance and participants were assigned a table number upon registration, to prevent the possibility of new siloes emerging in the workshop setting (if, for example, participants chose to sit with people they recognised or had previously worked with). However, this did mean that dynamics between stakeholders were unknown, and there was a risk of some individual voices overshadowing others, or power imbalances leaving some participants out of the discussion, or feeling uneasy. To counter this, we asked all workshop participants to ensure that all members of their groups had a chance to speak, and we placed at least one facilitator on each table to manage or steer the discussion, and invite particular contributions when necessary. The overall representation within the 130 contributors was mixed, and professionally diverse, but with very little involvement from large scale private businesses or profit-driven industries. Also, the community-focussed workshop attracted a self-selecting group of residents who were already likely to be involved in participatory events and processes. To gain richer or more comprehensive understanding of what matters in ‘thriving’ terms to under-served populations, a more purposeful and targeted approach to recruitment would have been needed (though that would have been beyond the scope and resource of this exercise, where ‘breadth’ was key).

The equal value attributed to different kinds of evidence, knowledge traditions and understanding of place by the groups (be it academic, strategic, practical/operational, or lived experience) was experienced as positive by participants in several of the workshops. Strategic responsibility and operational insight are also equally valued in discussions around this framework, particularly when discussing barriers to progress and implementation that those working ‘on the ground’ were more readily able to identify. The ‘levelling’ between participants was most notably the case when all participants were recruited from the same organisation (here, City Council staff), when the size of the organisation, separation of departments, and hierarchies of seniority would mean that direct meetings or personal interactions between these professionals were unusual. An important part of the Doughnut approach involves collaboration and partnership between multiple actors in the system, in order to break down silos, build coalitions, and integrate multi-level support, buy-in and commitment to action. The workshops proved to be an important mechanism for bringing together disparate stakeholders, each with influence on different Doughnut components. In terms of equity between the dimensions, participants found it more straightforward to answer the ‘local’ questions about thriving, with substantially more probing needed from facilitators on the global lenses (global-social in particular).

When asked what they enjoyed most about the workshop, feedback from participants highlights the value of working in an interdisciplinary way, for example

Interacting with other stakeholders across sectors and industries.

People coming from different backgrounds to deal with the cause.

Very diverse inputs from across sectors.

Diverse voices with different knowledge sets and experiences.

Cross-system synergies were specifically highlighted through the use of the Portrait framework. When taken as a full picture of multidimensional wellbeing, the co-created Thriving Definitions (‘purposeful activity models’ in SSM terminology) are broad, ambitious, and suggest multiple points of interconnection and/or cross-system support. For example, ‘transport’ features in each of the four lenses (either in core definitions or in the enabling factors). Taking the four different perspectives allows for a comprehensive understanding and assessment of what a fully holistic approach to Glasgow’s transport system might entail. Specifically, in the Local-Social lens, ‘mobility’ is a full dimension in its own right, but transport also features as an enabling factor in creating access to decent employment opportunities. In Local-Ecological, switching to clean or active transport would support the ‘cleanse the air’ definition, and decarbonising transport systems overall would reduce pressure on the Global-Ecological dimensions of ‘climate change’ and ‘ocean acidification’. Reducing transport emissions is also identified as a way of minimising Glasgow’s harmful Global-Social implications, through their impact on floods, crop failures, and sea level rise.

Some workshop discussions also highlighted the more challenging issues of trade-offs and negative consequences between dimensions, such as the potential conflict between creating a less ecologically harmful agriculture and food system, and providing nutritious, affordable food for all Glaswegians (who have had historically poor diets and associated health problems). Trade-offs were also highlighted for issues such as providing cheap school uniforms, and the lived experience of communities whose environmental expectations for new planning developments were able to be disregarded due to policy loopholes. One limitation of using the Doughnut Framework to create the full set of 44 Thriving Definitions has meant that we have been unable to fully capture or use the richness of qualitative insights that emerged from these workshops, given the scope of this research (at least at this foundational stage) on breadth and big-picture thinking rather than detail.

**Fig. 5:**
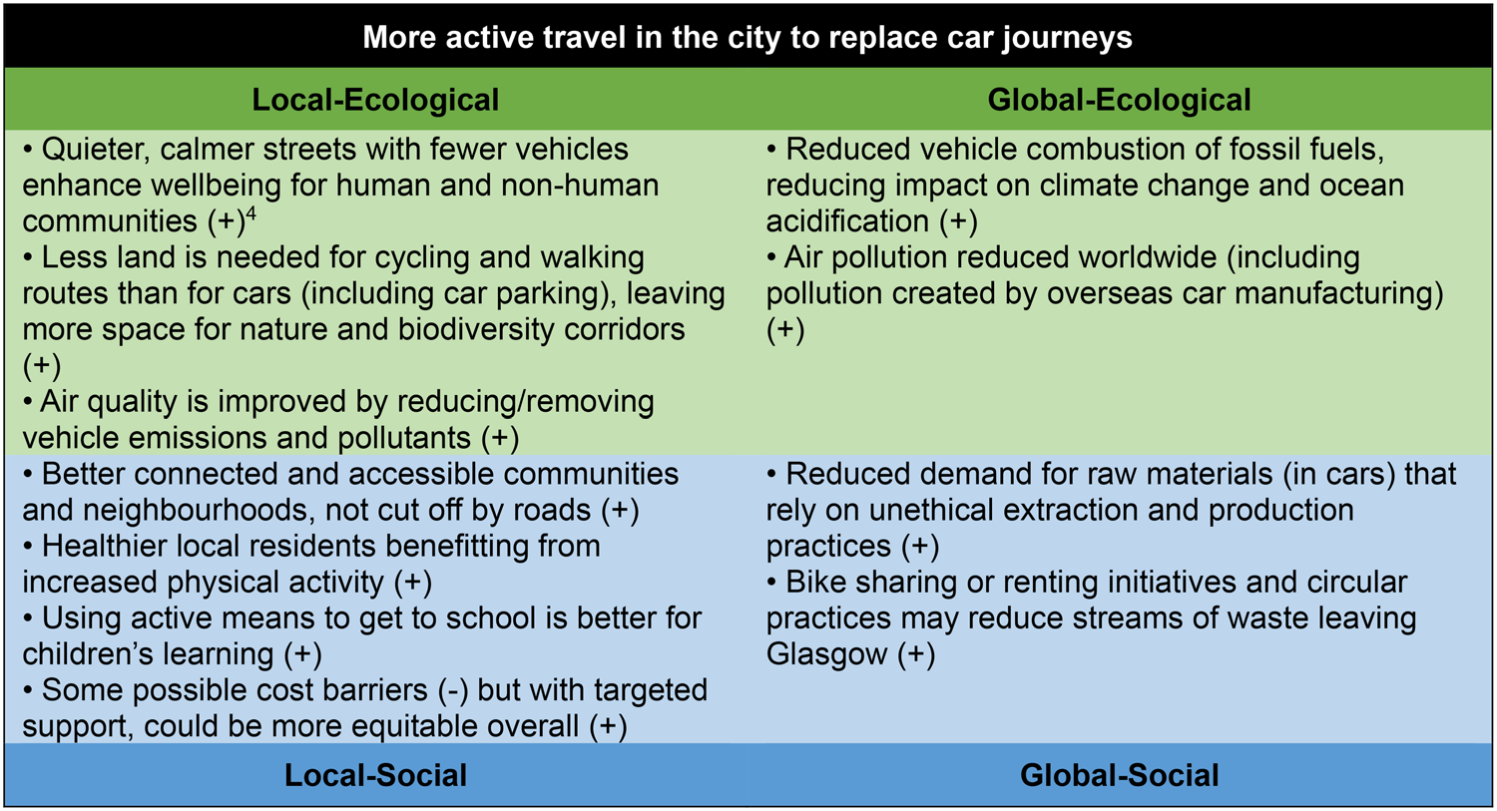
Examples of the four-lens view on priority policy areas in Glasgow. ^4^(+) suggests the action will be positive i.e. take Glasgow closer to the Doughnut’s boundaries (‘safe space’). (-) suggests the action will be negative i.e. take Glasgow further away from the Doughnut’s boundaries (‘safe space’)

**Fig. 6:**
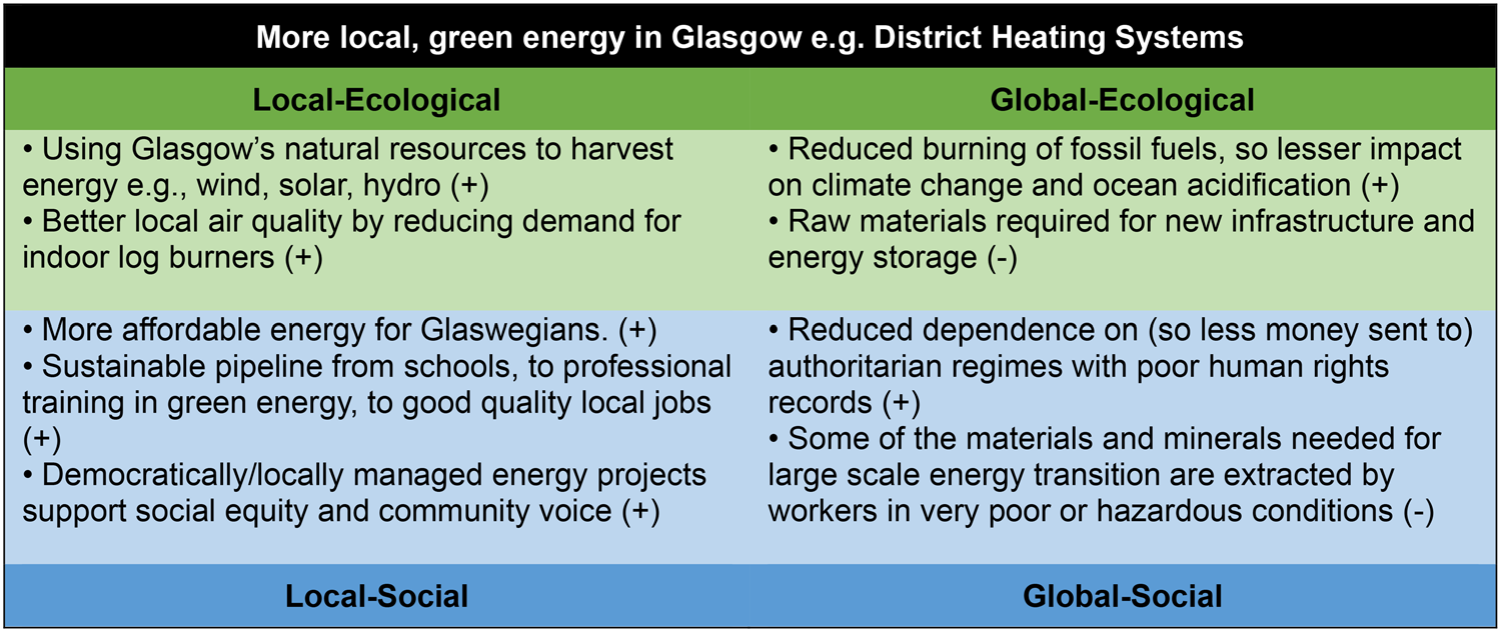
Examples of the four-lens view on priority policy areas in Glasgow.

**Fig. 7:**
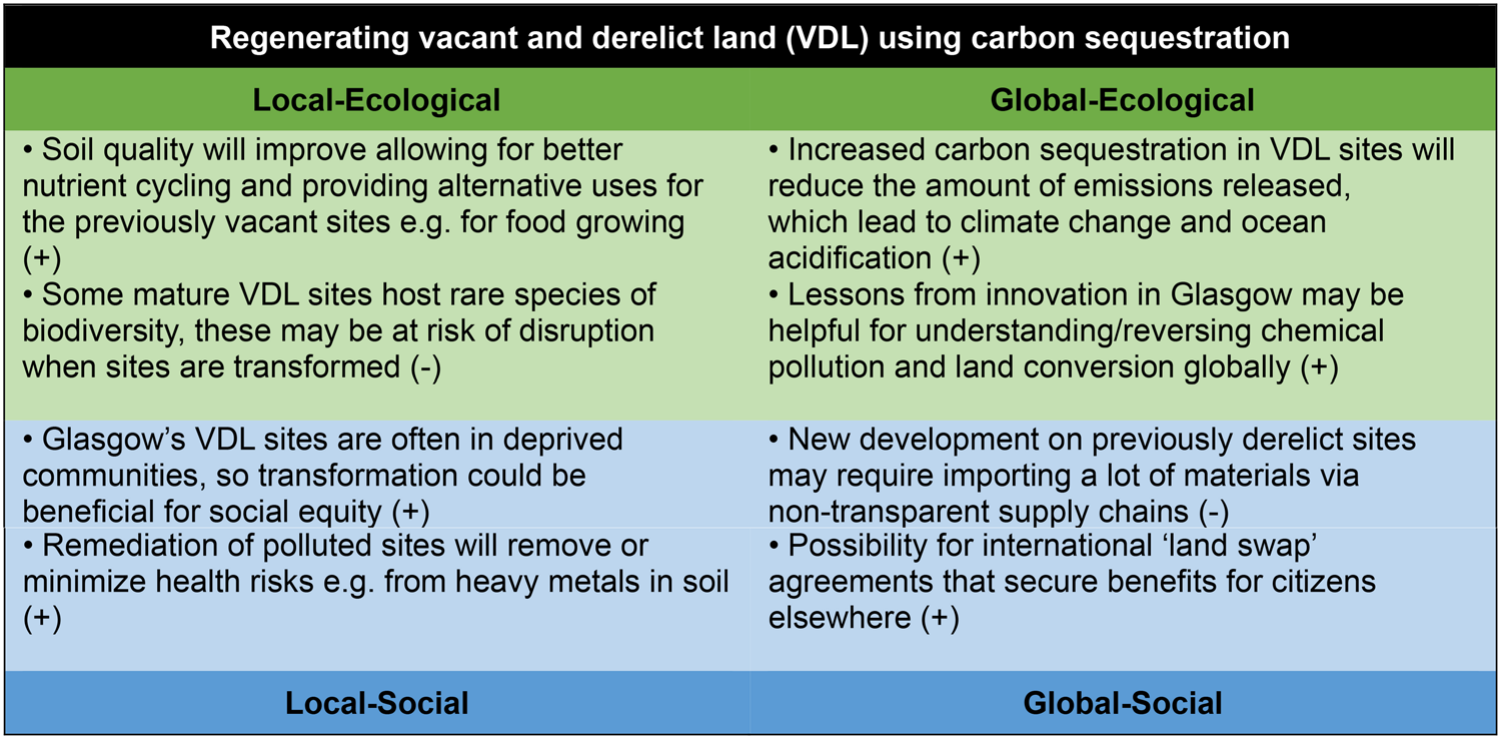
Examples of the four-lens view on priority policy areas in Glasgow.

The framework also enabled workshop participants to consider policy priorities or ambitions from the interconnected wellbeing perspectives (see Figs. 5, 6 and 7), and to compare possible co-benefits and trade-offs using the four-lens structure. Through this framing, the potential co-benefits and possible unintended negative consequences are highlighted, enabling decision-makers to consider systemic effects that may be felt elsewhere in the system. The potential of a systems approach to both maximise co-benefits (where action in one area triggers a positive impact in another) and minimise trade-offs (where benefits for one goal may result in negative consequences for another) was highlighted through this exercise and enabled by the structure of the Portrait framework.

### Agreeing and formal adoption of thriving definitions by the city council and launch

Following the workshops, an iterative process of co-production between the research team and council officers was undertaken. The researchers presented draft versions of all 44 definitions (and ‘what this could look like’) to a focus group of council officers, and through a series of in-person and online meetings the wording of each was finessed and collaboratively agreed. Some challenges arose here when seeking wording that was deemed to be politically palatable but also stayed true to the intent of the stakeholder participants. Civil servant leadership proved to be valuable in quelling doubts here, with senior governmental staff demonstrating they were prepared to uphold the results of the workshops. The process was designed to ensure the wording of the definitions was consistent throughout, and that they would resonate and be applicable across multiple departments. Each stage of the process secured additional buy-in and support from different levels of governance and policymaking, culminating in the approval and adoption of the thriving definitions first by Glasgow’s Net Zero Committee and then the City Administration Committee (the highest level of Glasgow City Council’s governance). While endorsed at the highest level, another challenge throughout the process was that we were not able to engage with all departments across the council equally, and there is likely significant work still to be done to socialise these ambitions more widely. A report for policymakers was launched in November 2023 (Hjelmskog et al. 2023), with the public support of the Leader of Glasgow City Council, who also endorsed the definitions and the Doughnut Economics framework at a high-profile launch event attended by cross-system, multi-sector stakeholders. The outcomes from this Portrait process will therefore lay the groundwork for the next steps of the soft systems approach, which will involve formulating policy pathways that are both ‘desirable’ and ‘feasible’, followed by taking iterative action to improve upon the situation in ways that align with the holistic Thriving vision.

## Discussion

In Glasgow, the Doughnut Economics framework has proven applicable in two significant ways: firstly, for structuring whole-system thinking around a complex socio-ecological policy area in a way that advances holistic understanding of the issue(s), and secondly, in providing a ‘compass’ for vision and ambition setting that focusses different city actors around a consistent, synergistic end goal. The resultant thriving vision represents the multi-dimensional contributions of city stakeholders from a wide range of social and/or ecological backgrounds, and the Doughnut framework was effective at condensing and summarising the views of diverse groups around a coherent ambition, which resonates in multiple professional and personal contexts. It also highlights the co-benefits and trade-offs that might accompany decisions, which is important at city and regional levels of governance (Jennings et al. 2020), although it remains to be seen whether the framework will enrich our understanding of the complex interlinkages and dynamics within the city system over time. Our experience of using the framework supports arguments that in comparison to other ‘beyond GDP’ approaches, Doughnut Economics has been characterised by its emphasis more on grand ambitions and framing of overall goals than specifics regarding means of arrival (Crisp et al. 2023). While it is a suitable framework for establishing this end state, we argue that it may be necessary to combine the Portrait methodology with complementary, formal approaches to defining and implementing effective change mechanisms and action pathways (for example, a Foundational Economy approach) (Wahlund and Hansen 2022).

The Portrait methodology led to a comprehensive suite of ‘conceptual models’ for Glasgow’s future success. The ‘grand vision’ outlined in the 44 thriving definitions (produced by the significant holistic/systems thinking analyses taking place in the workshops) has been positively embraced by stakeholders across different sectors and has received senior political approval (Council 2023). This is promising in terms of creating new paradigms and of the importance placed on worldviews as a key leverage point for intervening in a system (Meadows and Sustainability 1999, Waddock 2020) and of the power of alternative narratives (Friel 2022). These transdisciplinary, pluralistic approaches are necessary for reaching actionable and implementable solutions to 21st Century challenges (Pachoud et al. 2023). In the USA, siloed thinking, false separation of interconnected issues, and lack of exposure to diverse views has been shown at the national level to be associated with ‘pluralistic ignorance’ and beliefs that widely supported climate adaptation/mitigation measures are in fact unpopular, which is likely to be holding back or slowing the transformation to sustainable solutions (Sparkman et al. 2022). The building of conceptual models here may prove to be the initial step towards achieving policy coherence, or ‘multisolving’ interconnected challenges (Sawin 2020). These Thriving definitions will lay the groundwork for defining and deciding upon action pathways that are considered both ‘desirable’ and ‘feasible’ across multiple perspectives, which is crucial for a SSM (Checkland et al. 2020). As an applied study of Doughnut Economics and City Portraits, this paper contributes to a growing evidence base on exploring this framework in particular geographical places and policy settings (Schmid 2025, Savini and Post-Growth 2024, Thompson et al. 2024).

What is less evident from the initial process is how well the framework will support these subsequent stages of implementation and driving tangible action(s) based around this Doughnut ambition. The different but mutually reinforcing ‘roadblocks’ to achieving sustainability are conceptual (worldviews), institutional, and technological (Beddoe et al. 2009). While this paper reports only on the initial stage of this evolutionary cycle of learning, we anticipate potential barriers in translating the whole-system vision into agreed, resourced, implementable pathways to action, that are able to overcome common governance challenges (including managing complex systems, ensuring goal coherence across different spatial and temporal scales, and navigating power dynamics, inequalities, and trade-offs) (Turner and Wills 2022, Friel et al. 2022). Workshop participants were quick to highlight where progress would be dependent on national policy decisions, or significant resource commitment from those in charge of higher budgetary tiers. For example, achieving widespread housing retrofit across Glasgow’s complex, multi-tenure tenement block system, and managing the associated costs and responsibilities, will likely require comprehensive tenement law reform (Gibb 2022). Action will also be required from all sectors of society to achieve these ambitions, including the private sector, which is likely to be captured by interests that work to protect their profits, and thus may be reluctant to transform (Friel 2022, Mialon 2020).

Challenges were highlighted around the necessary monitoring, evaluation and learning. This action is required urgently, due to the nature of the crisis we face, yet the evidence base for ‘what works’ will necessarily be developed during this ongoing process of solutions-focussed research. Stakeholders will therefore be asked to accept a degree of uncertainty. Identifying ‘criticality’ (the most critical moment or factor) in terms of social tipping processes is usually only possible in hindsight (Winkelmann et al. 2022), although Glasgow has an opportunity for ongoing learning, as proposed solutions are implemented and evaluated in real-time. We therefore anticipate challenges around the City’s ability to measure progress and track meaningful metrics against these ambitious understandings of thriving. Current city measurement practices rarely account for value-based indicators that go beyond GDP or GVA, yet most examples of ‘thriving’ suggested during the Portrait process illustrate social or environmental value. It has proved difficult in other political contexts (even when actors are willing) to shift the focus from growth-oriented metrics to ones that meaningfully reflect wellbeing (Hayden and Dasilva 2022), though this is a growing policy interest area (MacLennan et al. 2021, Stickland and Beyond 2023). It will be necessary to monitor and assess the extent to which the ambitious perspective on thriving is adapted or incorporated into pathways or defined mechanisms that can be meaningfully evaluated. As the research programme continues, the collaborative relationship and process of co-creation with Glasgow’s policymakers may provide a breadth of expertise in deciding how to effectively socialise the ambitions and identify the right indicators of progress.

The Doughnut framework does provide some unique strengths in comparison to other wellbeing approaches, which were highlighted by the process of engaging with stakeholders in Glasgow. The framework is intuitive (once explained) and allowed for insightful discussions and creative ideas to surface. The identification of important policy synergies and trade-offs, beyond simplistic linear effects, suggests that the Doughnut helped participants name emergent effects that go further than their usual departmentalised or siloed ways of thinking. For example, the challenges associated with providing affordable school uniforms for Glasgow’s children in ways that minimise the global ecological and social footprint associated with mass-production of cheap clothing (Hjelmskog and Glasgow’s City Portrait Workshops Begin 2022). A further example is the connections drawn between securing affordable, renewable energy for Glaswegians that could have the added benefit of reducing dependence and therefore trading with authoritarian regimes that supply fossil fuels (Hjelmskog and Workshop with Multi-Sector 2023). Something important that the four lens Doughnut Economics framework does offer spatially, which is often missing in city settings, is the ongoing focus on global relationships and the city’s impact and influence on wellbeing outcomes for populations worldwide. Although stakeholders tended to have less well-developed knowledge or available data for these dimensions (particularly the Global-Social lens), it structures a more comprehensive understanding of this in the portrait methodology: matching local aspiration with global responsibility. This supports a core principle of the Sustainable Development Goals, particularly for countries that have contributed most to the pressure currently placed on planetary boundaries (Morton et al. 2019).

### Challenges and limitations of the approach/framework

The application of the Doughnut framework at the city scale creates some challenges spatially, both between the city and its wider geographical context, and internally, between different city neighbourhoods and communities. Firstly, the multiple tiers of governance that influence or direct city outcomes are not all under the city’s direct sphere of influence. Achieving the vision set out in the City Portrait will require many potential interventions or use of policy levers that the city itself does not control, and others have remarked that Doughnut Economics/Wellbeing Economy approaches can be ‘ambiguous and loose’ regarding the geographical scale(s) of action and influence that are necessary for reaching the overall goal (Crisp et al. 2023). Spatial differences (within the city) are also not easily accounted for in the portrait model. Although the Local-Social lens specifically names ‘social equity’ and ‘equality in diversity’ as key components of thriving, the other definitions are read as applicable to the whole city. This makes sense for visualising an equitable end state, but the necessary pathways to reach this state of thriving are likely to be very different for different neighbourhoods, particularly in cities such as Glasgow which experience high social, health, and economic inequalities (Schofield et al. 2021). It is also possible that even a maximisation of one city’s potential in terms of Doughnut-related levers, if it were to fit the ‘typical temporal and spatial scales of social tipping elements’ that commonly occur at the national or sub-national level, would be inadequate by itself to tackle the global threat of climate breakdown (Winkelmann et al. 2022). The potential of cities to act as drivers of change (Roberts and Mukim 2023) and the influence of multiple cities working in parallel may, however, have the potential to spread or amplify their impacts to a level that influences global trends and further reaching spatial scales or international agreements.

This paper looks at the potential of ‘Downscaling the Doughnut’ for whole system thinking and framing rather than the potential for quantitative analysis and assessment of place that is also key, particularly for measuring and monitoring progress. This paper does not go into detail about the process of analysing Glasgow’s current performance in terms of the doughnut’s boundaries, the quantitative ‘downscaling’ process, and challenges of applying a robust and consistent method of measurement across such a diverse range of dimensions. This formed part of the desk-based research and we found that a heuristic, non-standardised approach was the only way to apply existing data/indicators to the framework. There are widespread challenges in reporting progress on the Sustainable Development Goals (Beaudry and Alvarado 2023), and previous research has argued that the quantitative downscaling process of Planetary Boundaries to a local context has led to either the global meaning or local characteristics of a place being lost (Ferretto et al. 2022). The next stages of this research intend to use the thriving definitions to take a fresh look at both *what* we should be trying to measure in terms of success, (i.e., standardised metrics and indicators), and to think about *how* we systematically link the relationships between and among dimensions in a consistent way that enables dynamic analysis of trade-offs between conflicting targets subject to finite resources and constraints, providing valuable insights for decision making. This forms part of a rich research agenda combining hard and soft systems methods, qualitative and quantitative assessment, and engaging with both top-down and bottom-up approaches.

## Conclusion

In conclusion, the Doughnut Economics framework shows significant potential in terms of its framing power. The use of this framework with stakeholders has highlighted its ability to transcend diverse or siloed mindsets and working practices, both in terms of thinking more holistically about the current system, and for setting future goals that are aligned with its principles. It is significant in not only *framing* the social and ecological value of the system, but putting them at the *centre*, which is a departure from more traditional economic assessments of ‘value’. What remains unclear is the extent to which it will or can lead to implementable action pathways, and identifying meaningful indicators of progress. Future research needs to address these challenges, in order to link the broad, future-oriented visioning that the Portrait process facilitates, with the real-world tensions that accompany the allocation of limited resources, managing trade-offs between competing priorities, and the power struggles or imbalances that are part of multi-level models of governance (particularly in cities and city-regions within a devolved UK nation such as Scotland). It may also be limited in its potential to achieve transformative change when used in isolation, suggesting a need to think carefully about additional approaches to combine it with, both for its implementation and its evaluation.

**Table 2:** Results

Local-Social (How can all the people of Glasgow thrive?)		
Dimension	Definition of ‘thriving’	What this could look like
Food	Everyone in Glasgow has affordable access to nourishing and sustainable food. There is no food poverty, and no need for food banks.	Local community food growing and food sharing initiatives are widespread, and healthy food options are more accessible and affordable than ultra-processed options.
Water	Everyone’s water requirements are met - clean, treated water for drinking, cooking and hygiene is readily available.	Water resilience is improved by not over-using clean, treated water (so needing fewer chemicals) for growing, gardening, washing, cleaning etc.
Health	All Glasgow citizens live long and healthy lives, and health inequalities are small. Glasgow’s environment promotes physical health, enhances wellbeing, and supports good mental health.	Everyone lives in a health-promoting environment, without contaminated soil and polluted land posing a health threat to residents, and with high quality green space to enjoy, clean air, active travel routes and plentiful cycle storage to allow for physical activity. Glasgow no longer has disproportionate levels of excess deaths from substance abuse or suicide.
Education	A well-funded education system (schools, colleges, universities) gives equal access to all Glaswegians the foundation for life-long opportunity and fulfilment. Everyone is Glasgow is supported to realise their potential.	All children are attending school and are able to benefit from learning without the additional challenges of poverty, such as hunger. Education providers help Glaswegians to become good climate citizens through a curriculum that emphasises teaching the value of biodiversity and a healthy ecosystem for human prosperity. Education providers actively support healthy meals, low-carbon travel and physical activity.
Housing	Homes for all residents, in all tenures, are affordable, secure, energy efficient, and free from health hazards.	Glasgow’s architectural heritage (tenements) is celebrated, but also possible to modernise/retrofit. Glasgow builds and regenerates more beautiful, low-carbon social housing. The interests of residents are prioritised over the profit of landlords. There is widespread city support for national level housing reform that provides sustainable methods of tackling rising Private Rental Sector inflation. Housing services and social landlords provide additional co-benefits to communities e.g. green spaces, local food growing, community pantries.
Energy	All of Glasgow’s energy needs are met from renewable sources. Everyone is able to afford their energy requirements.	Glasgow’s energy consumption (direct and indirect) is much reduced overall, in particular by combining energy efficiency measures with decarbonising heating, and reducing the consumption of high-consumption industries and individuals. More renewable energy infrastructure is locally owned and managed within Glasgow and surrounding communities, generating community wealth.
Connectivity	All citizens in Glasgow have access to fast, affordable broadband and are supported in accessing online communication networks and the internet. There are no digital inequalities by age, wealth or education status.	n/a – no further suggestions made in workshops
Mobility	It is easy, safe and affordable for Glaswegians and commuters to get around the city sustainably at all times of the day.	Glasgow’s compact, liveable neighbourhoods with access to key infrastructure and services are connected by a cheap, decarbonised, fully integrated public transport system that provides a regular service throughout the day and night. Glasgow is well connected to other cities and rural areas.
Community	Glasgow is a friendly and welcoming city. All Glaswegians have opportunities to take an active role in their communities, supporting vital community connection and services.	There are widespread community sharing initiatives and communal resources such as material exchange hubs and ‘libraries of things’, for tools, toys, and gardening equipment. More people have the ability take part in volunteering. There are plenty of community spaces available for use in all seasons, and opportunities for local communities to act as stewards of these spaces.
Culture	People in Glasgow enjoy a healthy ‘work-life’ balance with lots of opportunity for cultural, leisure and wellbeing-promoting activities that are both affordable and inclusive.	Our cultural assets such as museums and creative spaces contribute to wider city ambitions, such as job creation.
Income & Work	All Glaswegians have sufficient income from a sustainable/fulfilling source. There is zero poverty in the city.	Job growth areas are in sustainable industries such as renewable energy, culture, and wellbeing services. Long-term investment is made in the skills and training to support these good quality jobs. Glasgow invests in and supports (through tools such as procurement) alternative business models e.g. social enterprises and cooperatives. Glasgow residents are connected to high quality employment opportunities e.g. through accessible transport options and inclusive recruitment practices.
Social Equity	All Glasgow citizens are valued equally, and all Glasgow’s communities benefit from inclusive access to, and representation in, city spaces and institutions.	Dramatic improvement and investment in the fabric of historically poorer areas, improving access to and ownership of ‘The Commons’ across all communities. Glaswegians act as stewards of public resources for the benefit of future generations. VDL sites in deprived areas are regenerated to provide amenities (natural, cultural, social, economic) to those who most need them. Policy levers are employed to protect against gentrification. Patterns of intergenerational poverty are broken.
Equality in Diversity	Glasgow recognises and celebrates the diversity of its communities. All Glaswegians, regardless of background or culture, have equal opportunities and access to everything Glasgow has to offer.	The diversity of all Glasgow’s neighbourhoods is celebrated and reflected in our decision-making and services e.g. through different languages. Services and city spaces are used equally by all groups in society - ethnic diversity and gender equality - reflected for example in patterns of active travel.
Political Voice	Glasgow takes an inclusive and proactive approach to policy and decision making.	Our political processes are informed by more inclusive citizen participation and engagement, valuing all voices including quiet ones (of different ages and backgrounds too). Public spaces and tools such as citizens’ assemblies are used to encourage more community engagement, and our political leaders demonstrate that they can be trusted.
Peace & Justice	Glasgow prioritises and resources residents’ safety, peace, and protection.	Neighbourhoods are safe and peaceful in all parts of the city, all individuals feel protected, and trust that resources are available to keep them safe e.g. in parks and greenspaces.
**Local-Ecological (How can Glasgow be as generous as the wildland next door?)**		
Cleanse the air	All Glasgow citizens breathe healthy and unpolluted air, and pollutants are well below maximum statutory guidelines for health.	Glasgow has active travel infrastructure, Low Emission Zones, urban trees and plants in public, private and commercial spaces. Its emissions from construction, transport and industry are minimised. Glasgow uses its policy levers to support widespread capture of pollutants, at individual and industrial levels.
House biodiversity	Glasgow and its surroundings have abundant and diverse local populations of native species.	Biodiversity protection and restoration is properly resourced, and built in to all other planning priorities. Crucial pollinators and worms are protected. There are well-connected habitats and wildlife corridors. City parks and waterways are managed holistically, to join up wildlife habitats. Vacant and derelict land sites can be assessed for biodiversity improvement. This may include protecting some mature derelict sites that support rare species or encourage returning biodiversity. Adequate training opportunities are available to citizens of all ages to improve skills in managing green spaces for biodiversity. Rewilding is encouraged in the city, maintenance teams do not cut back grass verges, and several wildflower meadows are planted.
Store carbon	Glasgow stores more carbon in its trees, soils, greenspaces, and waterways than it releases. It maintains and protects its natural carbon sinks.	Heating, transport, and construction sectors are decarbonised, through use of Nature-Based Solutions, the use of net zero construction materials, and repurposing of industrial infrastructure for carbon sequestration. Glasgow supports the restoration of carbon sinks across the wider region e.g., peatlands, seagrass.
Cycle water	Glasgow recognises the value of clean water. It maximises opportunities for grey water recycling, rainwater harvesting, and manages wastewater sustainably. The rivers are free from pollution and support healthy biodiversity.	Glasgow makes efficient use of untreated water (needing fewer chemicals) for growing, gardening, washing, and cleaning. Green infrastructure, nature-based solutions, and sustainable urban drainage systems are widely used. Urban design practices embrace adaptation through rain gardens, flood plains, more permeable surfaces (to reduce pollutant run-off), and healthy river corridors that can also provide wildlife habitat.
Harvest energy	The Glasgow energy mix is harvested from renewable sources to benefit Glasgow residents and businesses. Glasgow pursues renewable energy generation and storage solutions.	Glasgow maximises opportunities to use solar, wind, water, and ground source heat for its energy, distributed through local heat networks. More approvals are granted for solar power and low-carbon energy infrastructure on publicly owned buildings. All new buildings fitted with solar panels and zero carbon heat sources. Glasgow explores new methods of generating and storing renewable energy e.g., kinetic energy, harvesting ‘Park Power’ from parks and open spaces.
Regulate the temperature	Considered design of the natural and built environment in Glasgow creates a balance of green, grey, and blue spaces that contribute to temperature regulation.	More urban green spaces are created to achieve a better balance between ‘urban’ and ‘natural’ spaces. Small spaces are maximised for plants and trees e.g. pocket street parks.
Build and protect soil	Soil in Glasgow is healthy, nutrient-rich, and fertile, which supports healthy biodiversity, food growth, and diverse green spaces.	Impermeable hard surfaces are minimised and replaced with permeable surfaces. Spaces are provided to support soil health through composting, regenerative agriculture, and hosting biodiversity. Vacant and Derelict Land sites need to be decontaminated and transformed in pursuit of soil health. Sites of Special Scientific Interest (SSIs) need to be conserved.
Enhance wellbeing	Green and blue spaces across Glasgow are plentiful, pleasant, accessible to all, and clean.	Glasgow’s open spaces are multi-purpose: they can be used for leisure and as ‘green gyms’; as community venues; and to provide connection with nature. The local community are engaged in the upkeep and stewardship of green and blue spaces, and benefit from its results. Support is provided to build capacity for volunteering. Currently polluted, vacant or derelict green and blue sites (including potential swimming spots) are prioritised for transformation, even if temporarily. The Glasgow Clyde Valley Green Network blueprint is enacted.
**Global-Ecological (How can Glasgow safeguard the health of the whole planet?)**		
Climate change	Glasgow has no net contribution to global greenhouse gas emissions.	Sectors most responsible for global warming are decarbonised and Glasgow has minimised the emissions embedded in its supply chains. Glasgow is not fed by industrial farming methods, especially for livestock with high Greenhouse Gas emissions, such as cows. Consumption levels of the heaviest emitters are curtailed, and specific sites responsible for very high emissions are targeted e.g. Glasgow Airport. Glaswegians eat primarily plant-based diets that create fewer Greenhouse Gases. Buildings are retrofit to Net Zero standards such as Passivhaus, to reduce our energy demand.
Ocean acidification	Glasgow contributes to protecting global oceans by having no net contribution to greenhouse gas emissions.	The sectors emitting most CO2 in Glasgow in 2022 - heating and transport - are fully decarbonised. Demand for CO2 burning activities is reduced overall, through investment in active and public transport, insulation and retrofit of buildings, and green local energy networks. Carbon Capture Storage is utilised on Vacant and Derelict Land sites.
Chemical pollution	Glasgow is committed to minimising its material footprint. Glasgow has a Circular Economy that maximises re-use of resources, minimises its use of harmful chemicals across sectors and supply chains, and is a global leader in the remediation of polluted urban environments.	Glasgow’s overall consumption is reduced, and the dominant consumerist culture is challenged by re-using, recycling, and repairing initiatives. Consumption of raw materials is minimised. Procurement policies and practices outlaw single-use products, pollutants, and items designed for obsolescence. Urban design and waste management is improved to prevent pollutants from entering the biosphere e.g. Sustainable Urban Drainage Systems (SuDS) protect the water system from microplastics. Glasgow’s procurement, construction, and waste management policies and procedures are transformed. Support is provided for alternative business models to keep materials and products circulating locally.
Excessive fertilizer use	Farming practices in Glasgow’s global supply chains are effective in minimising reliance on excessive fertilizer use. Glasgow supports and requires sustainable land use throughout its supply chains.	Glasgow’s surrounding farmlands utilise permaculture or regenerative agriculture methods. We support small-scale, local, food businesses (both growing and selling). Glasgow does not waste food. We use any food excess for compost to enrich local soil health. Subsidy or tax relief is provided to sustainable or regenerative food businesses. We can use previously vacant and derelict sites for growing food, cycling water and regenerating soil, in order to reduce dangerous nutrient loading elsewhere e.g. further upstream Glasgow’s rivers.
Freshwater withdrawals	Glasgow contributes to a sustainable and fair global water footprint.	The water footprint of Glasgow’s production and consumption is minimised. People in Glasgow adopt circular economy principles in their water consumption patterns. Supply chains are fed by sustainable practices in the food and textile industries. People eat predominantly plant-based diets, that are less water intensive than the production of meat. People in Glasgow have access to transparent, easy to understand information about how sustainable our overall use of water is, and the water footprint of our products. Communication campaigns inform residents about Glasgow’s water footprint, and the volume of water required for everyday items (clothes, food etc.)
Land conversion	Glasgow’s supply chain is built upon transparent and sustainable land use practices, ensuring responsible and accountable land management. Glasgow maximises its global influence to support restorative land use practices.	Glasgow makes use of urban allotments, vertical farming, and city rooftops, to reduce the amount of land used for agriculture, and allow more natural biodiversity to flourish. Food produced by industrial agriculture methods that cause deforestation is not imported into the city. Policy interventions incentivise good practice, penalise damage to natural landscapes, and empower communities to be involved in sustainable land management.
Biodiversity loss	Glasgow uses its political influence and convening power to facilitate awareness raising, evidence generation and debate around biodiversity solutions.	Glasgow carefully disincentivises supply chains and consumption practices that have negative consequences for global biodiversity, whether that is through pesticide use, CO2 emissions or waste management. Agriculture and fishing are less intensive, maintaining sustainable level of fish populations e.g. wild salmon. More urban allotments are used for food growing, leaving untouched land intact. Glaswegians understand the importance of biodiversity for human and planetary health through education and communication initiatives.
Air pollution	Glasgow supports and requires activity in the city and its supply chains to eliminate contributions to global air pollution.	Public and active transport is enabled throughout the city, with lots of connected walking and cycling routes. Highly polluting forms of transport (including air travel) are minimised. Policy and financial support is available for initiatives such as Liveable Neighbourhoods and Low Emission Zones, to disincentivise car use and promote active travel and more widespread use of public transport. Glasgow reduces its industrial emissions footprint by ending the production and consumption of single-use or short-life items (including products imported from around the world).
Ozone layer depletion	Glasgow avoids using ozone-depleting chemicals and gases, and contributes to the development of alternatives through research and development.	Raising awareness and education about the harms of ozone layer depletion, and which substances contribute to this. Support provided for suitable alternatives, and more ozone-friendly practices, such as more sustainable forms of transport in the city, strengthened by environmental regulation.
**Global-Social (How can Glasgow respect and support the wellbeing of people worldwide?)**		
Food	Glasgow supports a human rights approach to food for all.	Glasgow does not import food for consumption here at the expense of more distant communities. Glaswegians do not consume unsustainable amounts of food that global populations rely on e.g. quinoa, nor foods that cause deforestation e.g. beef, avocadoes. Local and low-carbon food production is supported to ease pressure on the global agricultural system. Information is made easily available about healthy and sustainable diets e.g. methods and benefits of switching to primarily plant-based food
Water	Glasgow does not use more than its fair share of water, either directly or indirectly (through imported goods).	Glasgow does not deplete global water supplies by importing unsustainable amounts of products that use water intensively, such as fast fashion. Glasgow does not pollute the wider water systems e.g. through antibiotics, nutrients in salmon feed, or microplastics. Material goods and clothing are reused and recycled, to reduce industrial burdens on global water supplies. Decontamination of vacant and derelict land sites, and tighter regulation and enforcement of environmental standards, reduces pollution in waterways.
Health	Glasgow acts to improve global health and wellbeing, and does not exacerbate existing threats to health and wellbeing through the working and industrial practices in its supply chains and recruitment practices.	Procurement practices are overhauled and supply chains coming into Glasgow do not involve dangerous or hazardous working conditions such as unsafe mining or child labour. Glasgow trains enough local citizens to fill essential health sector jobs, avoiding the tendency to recruit from other nations at levels that leave their local populations underserved. Food exported from Glasgow and its regions (such as fish) is free from harmful toxins e.g. heavy metals. The labels on products for sale in Glasgow are improved to offer trustworthy information about the conditions and wellbeing of workers in the supply chain.
Education	Glasgow supports and advocates for equal rights and access to education for all global populations.	Students worldwide are able to benefit from Glasgow’s high-quality universities. Opportunities for global collaboration support gender and social equity (lower international fees, more ways to participate remotely) and sustainability (not dependent on high-carbon transport e.g. flying) Student exchange programmes are established and extended to build worldwide solidarity between students and share multidirectional learning. Goods in Glasgow’s supply chains are not provided by child labour, which removes the opportunities for children worldwide to receive an education.
Housing	Glasgow does not contribute to conditions that threaten the stability and security of places and materials for global communities.	Glasgow reduces its contribution to climate change via CO2 emissions, which is causing floods, crop failures and rising sea levels in particularly vulnerable global communities e.g. The Island Nations. Direct and indirect Greenhouse Gas emissions in the most intensive sectors such as transport, agriculture, and heating, are minimised.
Energy	Glasgow uses and produces energy in ways that contribute to global Net Zero ambitions, and is considerate in its direct and indirect use of energy to minimise global energy consumption.	Glasgow is developing methods of green energy generation and storage that can be exported and shared worldwide, to reduce other nations’ dependence on extracting and burning fossil fuels. Glasgow’s material footprint is minimised, to reduce embedded emissions, and is not supplied by goods or products that are made using fossil fuel energy.
Income & work	Glasgow supports Fair Trade principles and good working conditions for all workers involved in its supply chains.	Glasgow does not import goods that are produced using forced labour, child labour, or hazardous working conditions. Initiatives are in place to minimise the need for new goods such as textiles that are likely products of poor working conditions e.g. setting up school uniform or sports uniform banks. Trade-offs are acknowledged and managed between Glasgow’s immediate need for certain goods (such as personal technology) and the poorly regulated countries and industries that supply them (including dangerous mining and factories).
Social equity	Glasgow takes responsibility as a Global North city to raise living standards worldwide, and reduce global inequalities.	Glasgow develops world-leading sustainable procurement practices and reduces its consumption of products from exploitative industries that erode social equity. Glasgow makes progress towards climate neutrality without increasing the burden on other countries e.g. through exporting waste. Glasgow does not fund or invest in practices or businesses that cause social or ecological harm e.g. through pension funds. Glaswegians are able to access better information and choice in relation to the social footprint of the goods and services consumed in the city.
Equality in diversity	Glasgow is welcoming to all people from all cultures and delivers healthy cultural inclusion. Glasgow’s activities do not support or uphold regimes with poor human rights records.	We challenge threats to equality, such as ‘hostile environment’ policies.
Community/networks	Glaswegians act as ‘global citizens’ to build community and solidarity worldwide.	Cultural and educational exchange programmes build strong networks and connection between Glasgow residents and global populations. Glasgow supports and welcomes climate refugees, and provides training and employment opportunities for newcomers to the city.
Political Voice	Glasgow sets a leading example of free, inclusive and intergenerationally just democratic engagement in political decisions.	n/a – no further suggestions made in workshops
Peace & justice	Glasgow ensures representation, inclusion, and protection of the rights of those most vulnerable to the effects of climate change.	Glasgow’s climate and ecological footprints are minimised, lessening its contribution to global instability, forced migration, and conflicts. Clean local energy reduces Glasgow’s dependence on imported fossil fuels e.g. gas. Short-term and long-term gains are carefully balanced e.g. Glaswegians who can afford to, are making certain sacrifices locally, to secure the long-term futures of people globally. Transparency and comprehensive reporting is insisted upon, from corporations and SMEs, about the footprint of their products and services. Glasgow supports the work of existing refugee organisations e.g. Refuweegee.

## Electronic Supplementary Material

Below is the link to the electronic supplementary material.

Supplementary Material 1

## Data Availability

Anonymised data can be made available on request.

## Footnotes

2 Glasgow publicly launched its participation in the Thriving Cities Initiative during its hosting of COP26, 2021. The project is a collaboration between C40 Cities, Glasgow City Council and the University of Glasgow to become a Thriving City, based on the ‘Creating City Portraits’ methodology (based on the example of Amsterdam)48.Initiative, T.C., *CREATING CITY PORTRAITS - A METHODOLOGICAL GUIDE FROM THE THRIVING CITIES INITIATIVE*. 2020, Circle Economy Foundation.

3 All tools provided by DEAL are licensed under the Creative Commons Attribution – ShareAlike 4.0 International Licence.
