# Supplementary figures and images for: Using doughnut economics to structure whole-system thinking with multidisciplinary stakeholders – a soft systems approach
Source: Urban Transform. 2026 Mar 18;8(1):10. doi: 10.1186/s42854-026-00093-1 (PMC13111525; doi:10.1186/s42854-026-00093-1)

**Supporting Information: The desk-based Portrait**


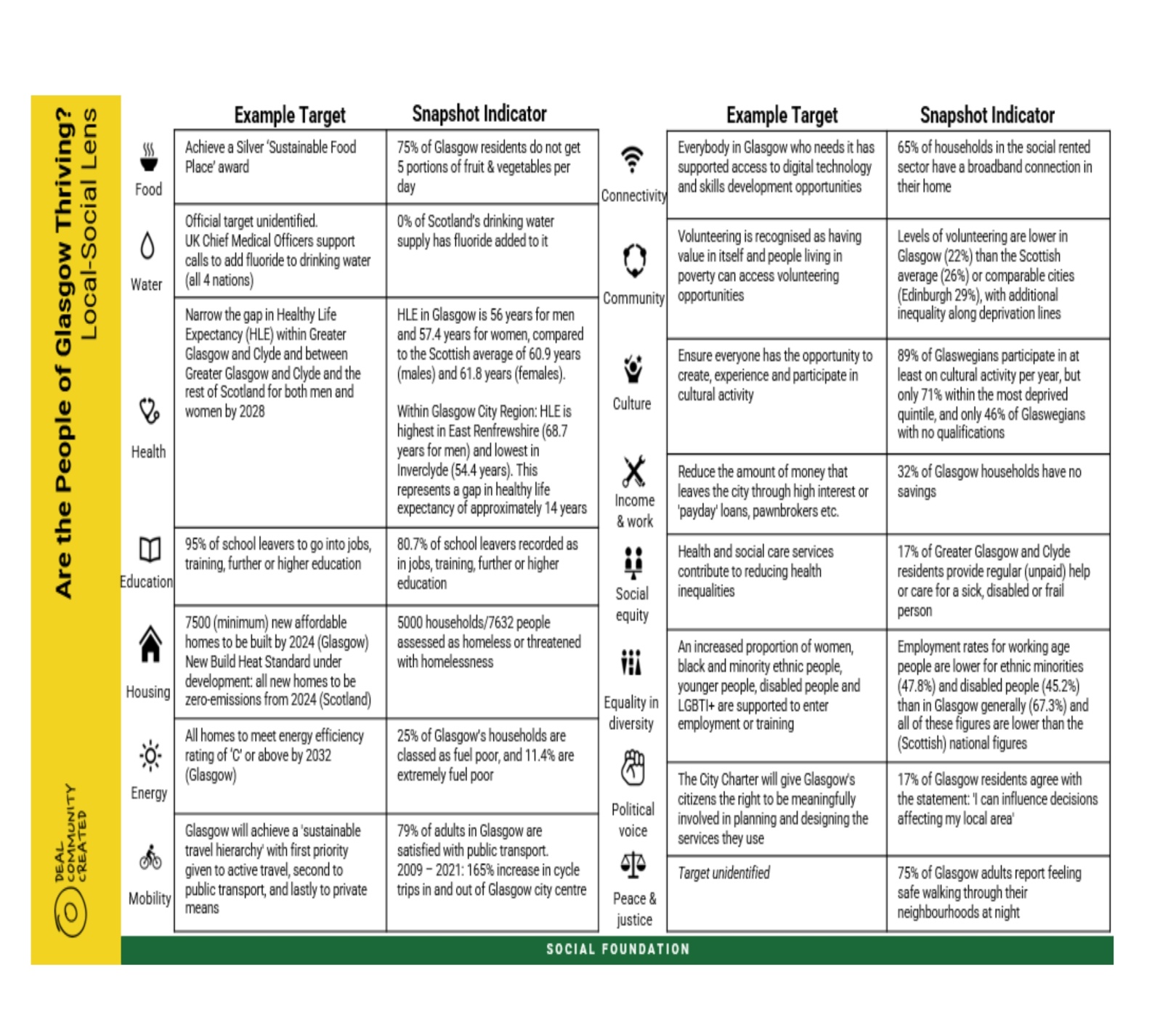


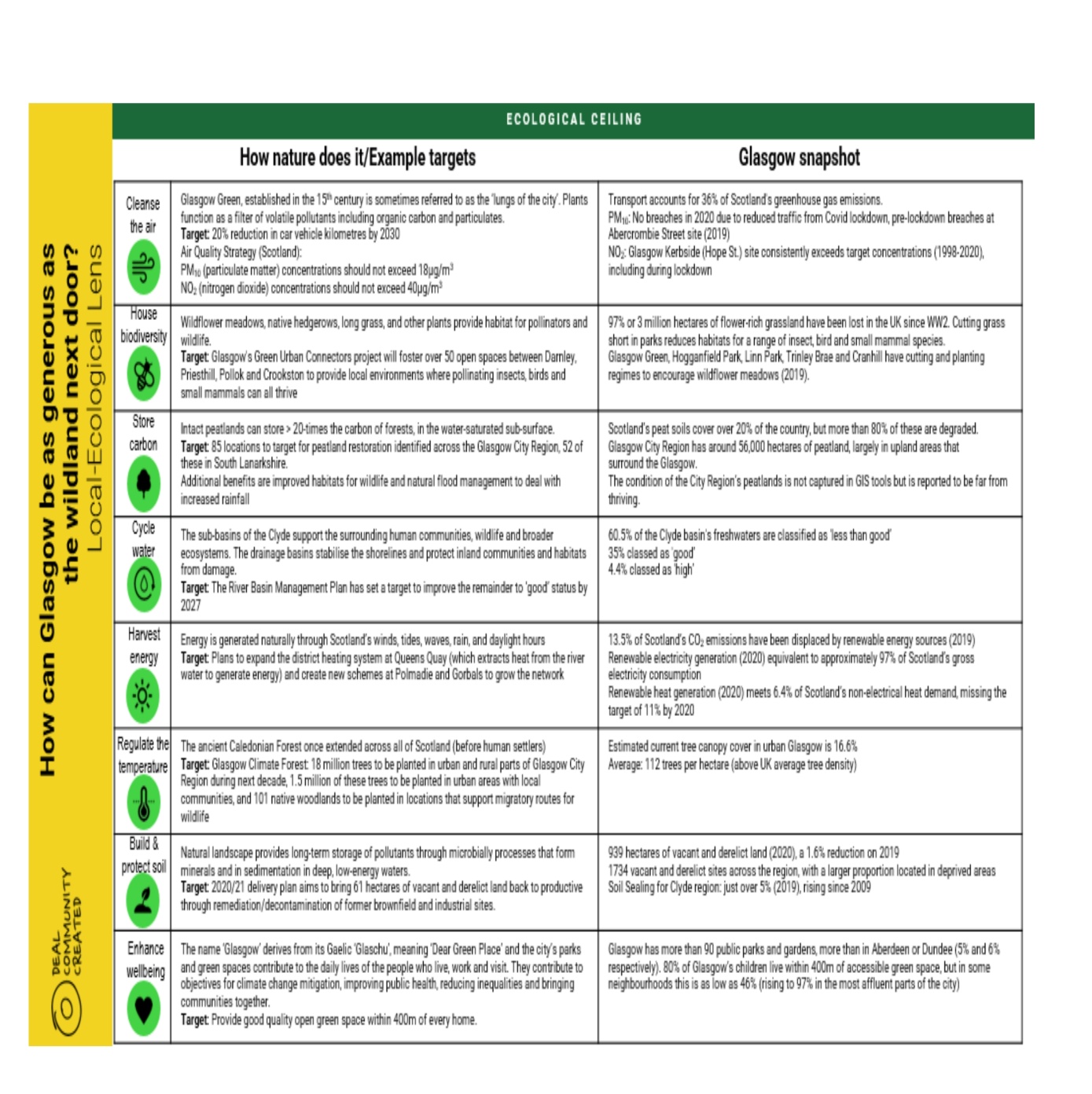


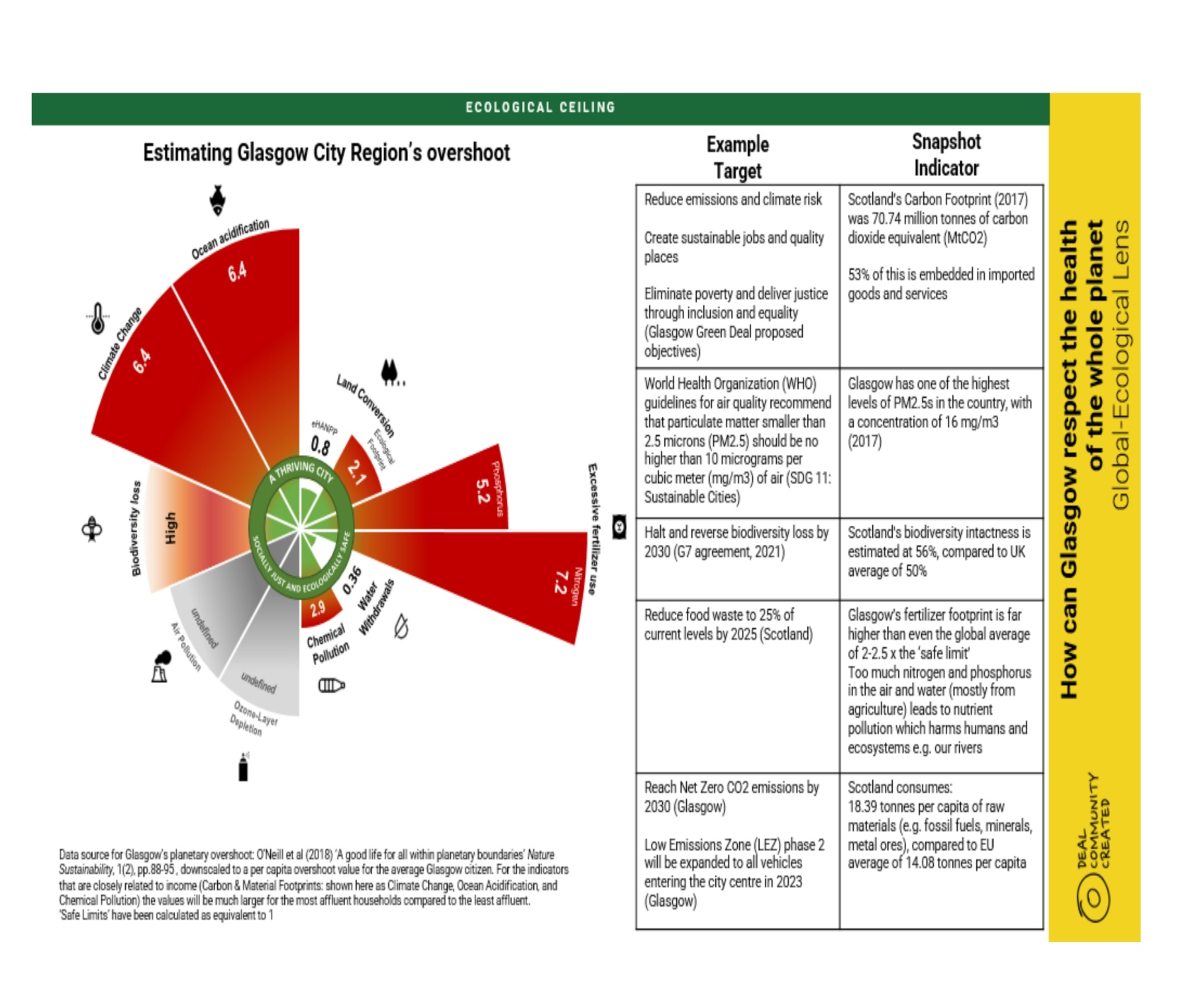


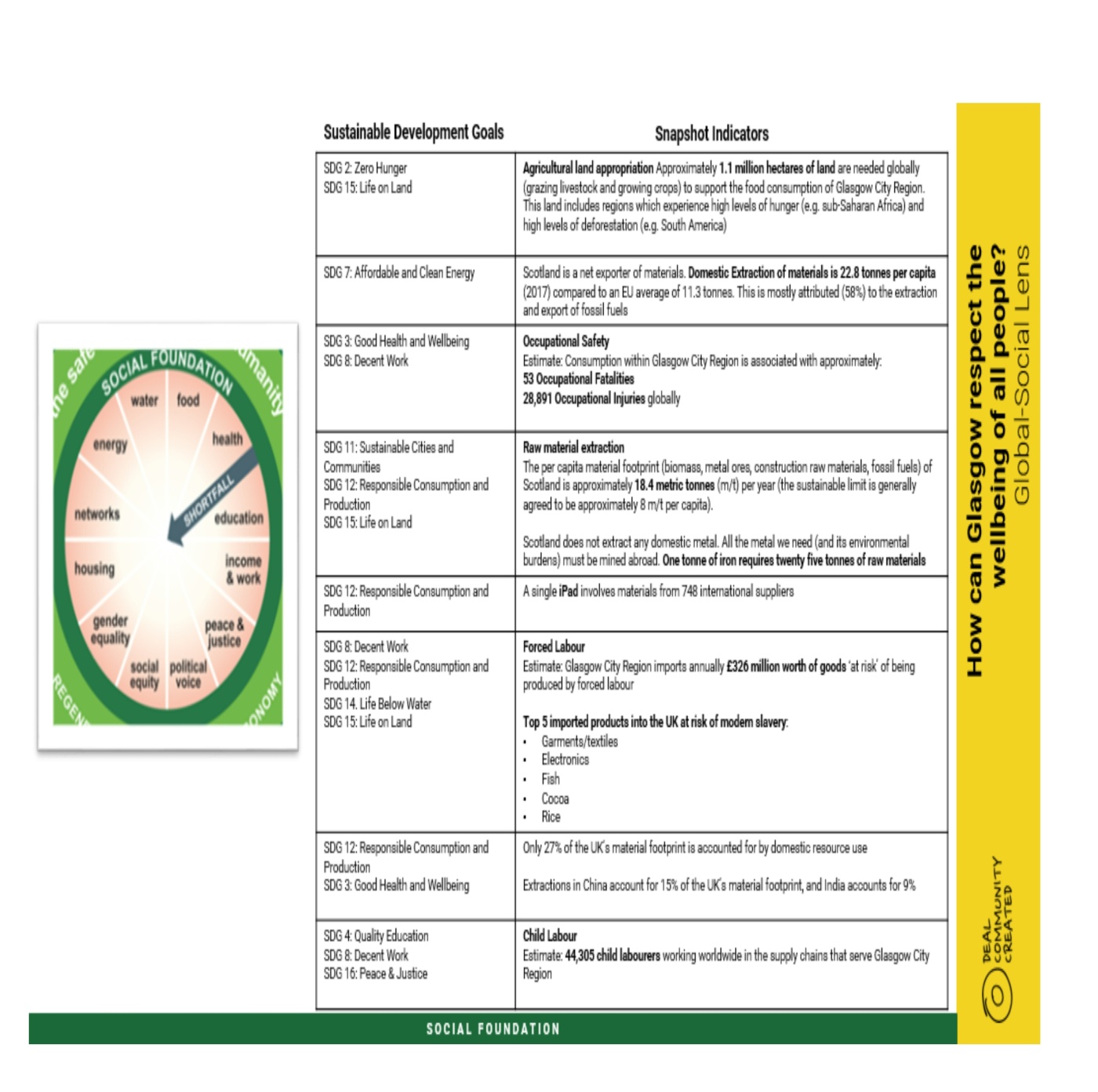

Supplement: Supplementary file 1 — Supplementary Material 1 [file 42854_2026_93_MOESM1_ESM.docx]
